# Current coffee consumption is associated with decreased striatal dopamine transporter availability in Parkinson’s disease patients and healthy controls

**DOI:** 10.1186/s12916-023-02994-5

**Published:** 2023-07-25

**Authors:** Chao Wang, Cheng Zhou, Tao Guo, Yeerfan Jiaerken, Siyu Yang, Xiaopei Xu, Ling Hu, Peiyu Huang, Xiaojun Xu, Minming Zhang

**Affiliations:** 1 Department of Radiology, The Second Affiliated Hospital, Zhejiang University School of Medicine, No.88 Jiefang Road, Shangcheng District, Hangzhou, 310009 Zhejiang China; 2 Department of Ultrasound in Medicine, Hangzhou Women’s Hospital, Hangzhou, Zhejiang China

**Keywords:** Parkinson’s disease, Coffee, Dopamine transporter, Striatum, Caudate

## Abstract

**Background:**

Coffee is the most widely consumed psychostimulant worldwide. Emerging evidence indicates that coffee consumption habit significantly reduces the risk of developing Parkinson’s disease (PD). However, the effect of coffee consumption on nigrostriatal dopaminergic neurodegeneration is still largely unknown. We therefore aim to investigate the role of coffee consumption in nigrostriatal dopaminergic neurodegeneration using dopamine transporter (DAT) imaging in PD and healthy controls (HC).

**Methods:**

A total of 138 PD patients and 75 HC with questionnaires about coffee consumption, and DAT scans were recruited from the Parkinson’s Progression Markers Initiative cohort. Demographic, clinical, and striatal DAT characteristics were compared across subgroups of current, former, and never coffee consumers in PD and HC, respectively. Furthermore, partial correlation analyses were performed to determine whether there was a relationship between coffee cups consumed per day and striatal DAT characteristics in each striatal region. In addition, the factors that may have influenced the loss of nigrostriatal dopaminergic neurons were included in multiple linear regression analyses to identify significant contributing factors to DAT availability in each striatal region.

**Results:**

PD patients had lower DAT availability in each striatal region than HC (*p* < 0.001). In PD patients, there were significant differences in DAT availability in the caudate (*p* = 0.008, Bonferroni corrected) across three PD subgroups. Specifically, post hoc tests showed that current coffee consumers had significantly lower DAT availability in the caudate than former coffee consumers (*p* = 0.01) and never coffee consumers (*p* = 0.022). In HC, there were significant differences in DAT availability in the caudate (*p* = 0.031, Bonferroni uncorrected) across three HC subgroups. Specifically, post hoc tests showed that current coffee consumers had significantly lower DAT availability in the caudate than former coffee consumers (*p* = 0.022). Moreover, correlation analysis revealed that cups per day were negatively correlated with DAT availability in the caudate in current consumers of PD patients (*r* =  − 0.219, *p* = 0.047). In addition, multiple linear regression analyses showed that current coffee consumption remained an independent predictor of decreased DAT availability in the caudate in PD patients and HC.

**Conclusions:**

This study demonstrates that current coffee consumption is associated with decreased striatal DAT availability in the caudate. However, the effects of caffeine on striatal DAT may fade and disappear after quitting coffee consumption.

**Trial registration:**

ClinicalTrials.gov, NCT01141023.

**Supplementary Information:**

The online version contains supplementary material available at 10.1186/s12916-023-02994-5.

## Background

Coffee is the most widely consumed psychostimulant worldwide [1]. After consumption, caffeine is rapidly absorbed through the gastrointestinal tract to the blood and then to the brain [2], leading to its short-term effects of causing alertness and reducing fatigue [3]. Long-term coffee consumption is associated with improved cardiovascular risk factors, improved asthma control, and lower liver disease risks and all-cause mortality [4]. Coffee consumption habit appears to attenuate the burden of some neurodegenerative diseases. Both retrospective and prospective epidemiological studies demonstrate that coffee consumption habit significantly reduces the risk of developing Parkinson’s disease (PD) [5–8], which is the second most common neurodegenerative disorder that affects 2–3% of elderly people > 65 years old worldwide [9]. Neuronal loss in the substantia nigra which causes a deficit of striatal dopamine and intracellular protein (α-synuclein) accumulation that leads to the nigrostriatal pathway impairment are the neuropathological hallmarks of PD [10]. The action of caffeine on the dopaminergic system is responsible for enhancing motor activity and exerting an antidyskinetic effect [11, 12]. There is evidence that coffee consumption is associated with reduced brain functional connectivity network related to somatosensory, motor, and emotional processing [13]. However, the effect of coffee consumption on nigrostriatal dopaminergic neurodegeneration is still largely unknown.

Dopamine transporter (DAT) is expressed in the dopaminergic neurons and clears the free dopamine released into the synaptic cleft [14]. In PD, the loss of dopaminergic neurons results in a substantial reduction of the DAT binding and dopamine levels [15]. Evidence indicates that caffeine acts as an adenosine receptor antagonist to interfere with dopaminergic transmission and enhance dopamine release with possible secondary reduced DAT availability [16]. In a mouse model of PD, exposure to caffeine significantly attenuates the loss of striatal DAT [17]. However, data are scarce from large, controlled human studies investigating the association between coffee consumption and striatal DAT availability in both PD patients and healthy controls (HC).

This study aimed to investigate whether coffee consumption was associated with the change in striatal DAT availability in PD patients and HC. Therefore, we systematically compared baseline striatal DAT availability across different coffee consumer subgroups (current, former, and never coffee consumers) in a large sample of newly diagnosed drug-naïve PD patients and HC enrolled from the Parkinson’s Progression Markers Initiative (PPMI) cohort.

## Methods

### Participants

Data used in this study were all obtained from the Parkinson’s Progression Markers Initiative (PPMI) database (http://www.ppmi-info.org) on July 1, 2022. PPMI is an ongoing observational, international, multicenter cohort study in a large cohort (ClincalTrials.gov NCT01141023). The study of the PPMI database aimed to identify clinical, imaging, genetic, and biospecimen biomarkers of PD progression. In the PPMI study, de novo PD patients were prospectively enrolled and exhibited presynaptic dopaminergic terminal loss as confirmed by DAT imaging. Study protocols and manuals are available on the PPMI website. The data sourcing of coffee consumption questionnaires was obtained from PPMI FOUND. PPMI FOUND was designed to enhance retention and minimize loss to follow-up of persons enrolled in PPMI, by providing a parallel longitudinal assessment method using Internet, telephone, and/or mail contacts with PPMI participants. The coffee consumption data were retrospectively collected. Although there were > 400 de novo PD patients in PPMI, only a relatively small subset of the PD and HC who completed questionnaires on caffeine consumption were included in this study.

**Fig. 1:**
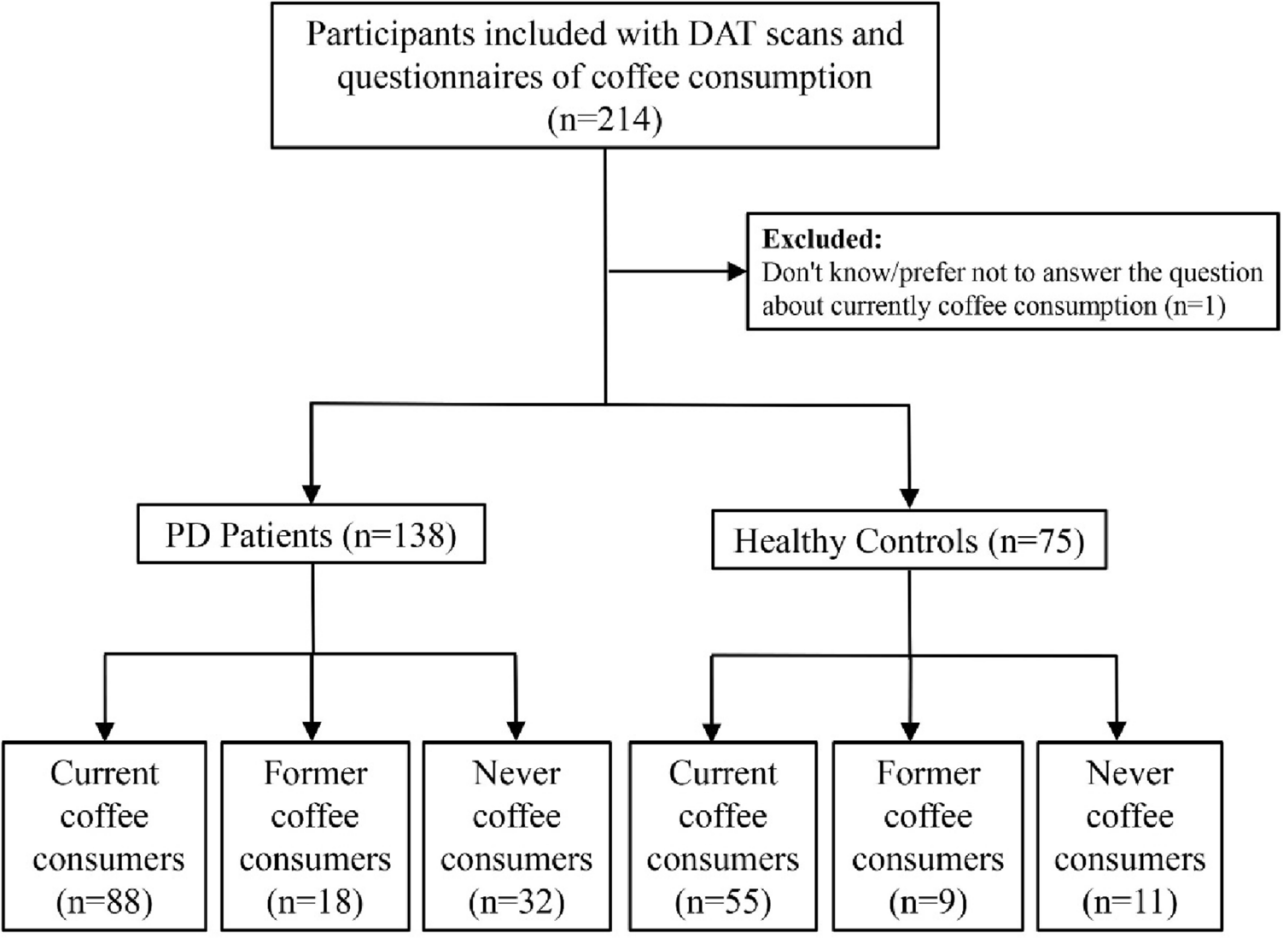
Flowchart shows participants’ enrollment

In all, 139 de novo PD patients and 75 HC with DAT scans and coffee consumption questionnaires were enrolled in this study. Coffee consumers were defined as people who have regularly drunk caffeinated coffee at least once per week for 6 months or longer and classified as “current” if they currently do and “former” if they no longer do. One PD patient who did not know/prefer not to answer the question “Do you currently drink caffeinated coffee?” was excluded. Therefore, this study finally included 138 PD patients and 75 HC. Specifically, PD patients consisted of 88 current consumers (PD-CC), 18 former consumers (PD-FC), and 32 never consumers (PD-NC), and HC consisted of 55 current consumers (HC-CC), 9 former consumers (HC-FC), and 11 never consumers (HC-NC). The participants’ flowchart is shown in Fig. 1.

### Clinical evaluation

All participants in the PPMI cohort received the standard test battery of assessments. In addition to the demographic variables (age, gender, and education), the clinical variables were collected, including the Hoehn and Yahr (H-Y) stages, the Movement Disorders Society Unified Parkinson’s Disease Rating Scale (MDS-UPDRS), the Montreal Cognitive Assessment (MOCA), the Geriatric Depression Scale (GDS), the Scale for Outcomes for Parkinson’s Disease–autonomic function (SCOPA-AUT), the State and Trait Anxiety Scale (STAI), the Questionnaire for Impulsive-Compulsive Disorders in Parkinson’s Disease (QUIP), the University of Pennsylvania Smell Identification Test (UPSIT), and the Epworth Sleepiness Scale (ESS).

### Striatal DAT processing

According to the PPMI imaging protocol, the baseline DAT images were acquired using [123I] FP-CIT single-photon emission computed tomography (SPECT) imaging at PPMI imaging centers. All PD patients were drug-naïve at baseline. Images were acquired using a gamma camera that is capable of performing SPECT imaging. SPECT scans were begun 4 h (± 30 min) post-radiotracer injection. The participant’s head was placed in the appropriate head holder and the field of view (FOV) encompassed the entire head to include the brain from most superior cortical regions through the inferior portion of the cerebellum. The participant was told of the necessity to avoid movements of the head and asked for her/his active cooperation. After the scan, SPECT data were sent to the Institute for Neurodegenerative Disorders for standardized processing. Striatal regions of interest (ROIs) were then placed on the target regions (caudate and putamen) and reference region (occipital cortex). Then, count densities for each striatal ROI were extracted and applied to calculate striatal binding ratios (SBR) for each striatal ROI. SBR was calculated as follows: SBR = (target region/reference region) − 1. Finally, the mean SBR of caudate and putamen was calculated. Detailed DAT processing procedures are available on the PPMI website (https://www.ppmiinfo.org/access-data-specimens/download-data/) and in published documents [18, 19].

### Statistical analysis

To assess the demographic, clinical, and DAT imaging characteristics between the subgroups, one-way analysis of variance (ANOVA) or *t*-test was used to compare continuous variables that did follow a normal distribution, the Kruskal–Wallis test was used to compare continuous variables that did not follow a normal distribution, and the *χ*^2^ test and Fisher’s exact test were used for categorical variables. The Kolmogorov–Smirnov test was used to assess the normality of the continuous variables. To determine whether there was a relationship between coffee cups consumed per day and striatal DAT characteristics, partial correlation coefficients were calculated for each investigated striatal region after adjusting for age and gender in the subgroups of current and former consumers, respectively. Furthermore, we included the factors that may have influenced the loss of nigrostriatal dopaminergic neurons in multiple linear regression analyses to identify significant contributing factors to DAT availability in the investigated striatal regions. This analysis was constructed using two models. In model 1, the independent variables included age, gender, coffee consumption (current vs. former/never consumers), and smoking history (current vs. former/never smokers). Model 2 included the independent variables from model 1 as well as age at onset and disease duration that were known to be associated with dopaminergic density in PD patients. Bonferroni correction was made to adjust for multiple comparisons of striatal DAT measurements. All statistical analyses were performed using the SPSS Statistics 26 software (IBM Corporation, New York). The significance threshold was set at *p* < 0.05.

## Results

### Demographic and clinical characteristics across subgroups of coffee consumption

Demographic and clinical characteristics of the three subgroups are summarized in Table 1. A total of 138 PD patients (47 women, 34.1%) and 75 HC (24 women, 32.0%) were enrolled. There were no significant differences in age and gender among the three subgroups of PD patients and HC. However, education years were lower in PD-NC than in PD-FC (*p* = 0.046). In addition, MDS-UPDRS, H-Y stages, MOCA, GDS, SCOPA-AUT, STAI, QUIP, UPSIT, and ESS were also compared across the PD or HC subgroups, and these clinical characteristics showed no significant difference.

**Table 1:** Demographic and clinical characteristics of PD and healthy controls subgroups

	PD-CC (*n* = 88)	PD-FC (*n* = 18)	PD-NC (*n* = 32)	*p* values	HC-CC (*n* = 55)	HC-FC (*n* = 9)	HC-NC (*n* = 11)	*p* values
Age (years)	60.1 ± 8.9	62.7** ± **10.0	57.7 ± 9.3	0.164	60.2 ± 10.4	58.7 ± 11.2	60.1 ± 11.7	0.928
Gender, ratio (F/M)	0.38 (24/64)	0.8 (8/10)	0.88 (15/17)	0.082	0.41 (16/39)	0.8 (4/5)	0.57 (4/7)	0.613
Education (years)	16.6 ± 2.5	17.4 ± 2.8	15.5 ± 2.2	0.032	17.3 ± 2.6	15.8 ± 2.0	17.1 ± 2.8	0.242
Coffee cups per day	2.0 ± 1.3	2.4 ± 2.2	NA	0.719	2.3 ± 0.9	3.1 ± 1.4	NA	0.065
Missing (number)	3	0	NA	NA	2	0	NA	NA
Current regular smokers, *n* (%)	1 (1%)	1 (6%)	1 (3%)	NA	0 (0%)	1 (11%)	0 (0%)	NA
Age at onset (years)	58.1 ± 9.2	61.3 ± 10.0	55.0 ± 9.5	0.068	NA	NA	NA	NA
Disease duration (months)	6.1 ± 5.5	7.5 ± 8.2	9.3 ± 9.5	0.279	NA	NA	NA	NA
H-Y stages	1.6 ± 0.5	1.6 ± 0.5	1.5 ± 0.5	0.765	NA	NA	NA	NA
MDS-UPDRS Part I	5.0 ± 3.6	4.9 ± 2.8	4.0 ± 2.7	0.442	2.9 ± 2.3	3.8 ± 3.2	3.0 ± 2.4	0.785
MDS-UPDRS Part II	5.8 ± 4.3	6.7 ± 4.2	5.2 ± 4.0	0.386	0.3 ± 0.8	0.1 ± 0.3	0.3 ± 0.5	0.657
MDS-UPDRS Part III	20.1 ± 7.5	21.8 ± 8.3	19.4 ± 9.2	0.564	0.9 ± 1.3	1.2 ± 1.1	0.6 ± 1.5	0.203
MDS-UPDRS Total Score	30.1 ± 11.6	33.4 ± 12.2	28.6 ± 12.3	0.376	4.1 ± 3.3	5.1 ± 4.0	3.9 ± 2.5	0.82
MOCA	27.3 ± 1.9	26.9 ± 2.4	27.8 ± 2.5	0.189	27.9 ± 1.0	28.4 ± 1.3	28.6 ± 1.3	0.217
GDS	1.9 ± 1.9	1.9 ± 1.5	1.9 ± 2.2	0.787	1.1 ± 1.7	1.0 ± 1.9	1.0 ± 1.2	0.73
SCOPA-AUT	8.6 ± 5.3	9.1 ± 5.0	6.8 ± 4.6	0.079	5.0 ± 2.9	5.8 ± 2.5	6.0 ± 3.6	0.496
STAI	61.7 ± 17.0	56.8 ± 17.6	61.6 ± 15.8	0.336	53.5 ± 11.7	61.7 ± 19.4	52.6 ± 13.8	0.435
QUIP	0.3 ± 0.6	0.4 ± 0.7	0.1 ± 0.4	0.238	0.4 ± 0.8	0.1 ± 0.3	0 ± 0	0.097
UPSIT	22.1 ± 7.8	24.6 ± 7.6	25.1 ± 8.0	0.151	34.7 ± 4.7	33.9 ± 5.9	33.6 ± 4.9	0.79
ESS	5.6 ± 3.3	6.0 ± 3.9	5.7 ± 3.3	0.968	5.4 ± 3.2	5.7 ± 3.9	6.3 ± 3.6	0.836

Three PD-CC did not know/prefer not to answer the question “cups per day.” Two HC-CC did not know/prefer not to answer the question “cups per day”

*PD-CC* current consumers of Parkinson’s Disease, *PD-FC* former consumers of Parkinson’s Disease, *PD-NC* never consumers of Parkinson’s Disease, *HC-CC* current consumers of healthy controls, *HC-FC* former consumers of healthy controls, *HC-NC* never consumers of healthy controls, *NA* not applicable, *H-Y* stages Hoehn and Yahr stages, *MDS-UPDRS* Movement Disorders Society Unified Parkinson’s Disease Rating Scale, *MOCA* Montreal Cognitive Assessment, *GDS* Geriatric Depression Scale, *SCOPA-AUT* Scale for Outcomes for Parkinson’s Disease—autonomic function, *STAI* State trait anxiety score, *QUIP* Questionnaire for Impulsive-Compulsive Disorders, *UPSIT* University of Pennsylvania Smell Identification Test, *ESS* Epworth Sleepiness Scale Score

### Striatal DAT availability across subgroups of coffee consumption

**Fig. 2:**
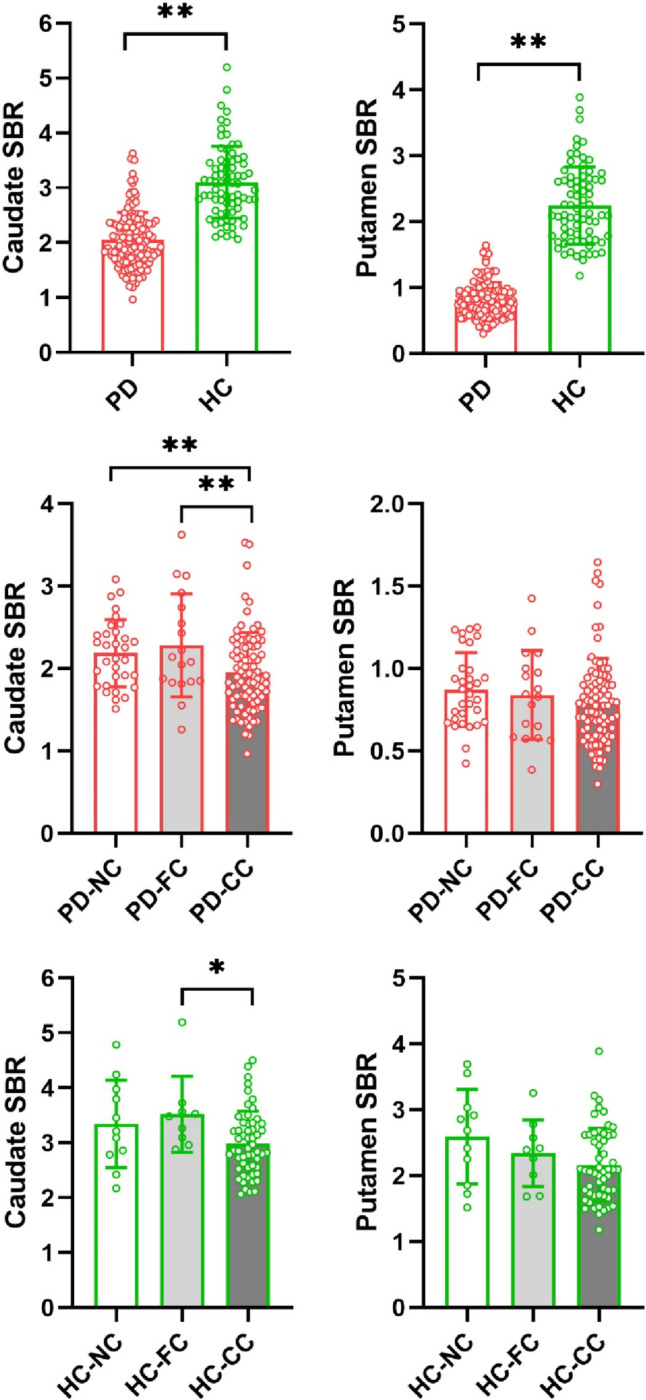
PD patients showed lower DAT availability in the caudate (*p* < 0.001) and putamen (*p* < 0.001) compared to HC. Current coffee consumers showed a tendency of lower DAT availability in the caudate than former/never coffee consumers in both PD patients and HC. In PD patients, there were significant differences in DAT availability in the caudate (*p* = 0.008) across three PD subgroups. Specifically, post hoc tests showed that current coffee consumers had significantly lower DAT availability in the caudate than former coffee consumers (*p* = 0.01) and never (*p* = 0.022) coffee consumers. In HC, there were significant differences in DAT availability in the caudate (*p* = 0.031) across three HC subgroups. Specifically, post hoc tests showed that current coffee consumers had significantly lower DAT availability in the caudate than former coffee consumers (*p* = 0.022) coffee consumers. PD-CC, current consumers of PD patients; PD-FC, former consumers of PD patients; PD-NC, never consumers of PD patients; HC-CC, current consumers of healthy controls; HC-FC, former consumers of healthy controls; HC-NC, never consumers of healthy controls; SBR, striatal binding ratios. **Bonferroni corrected; *Bonferroni uncorrected

**Fig. 3:**
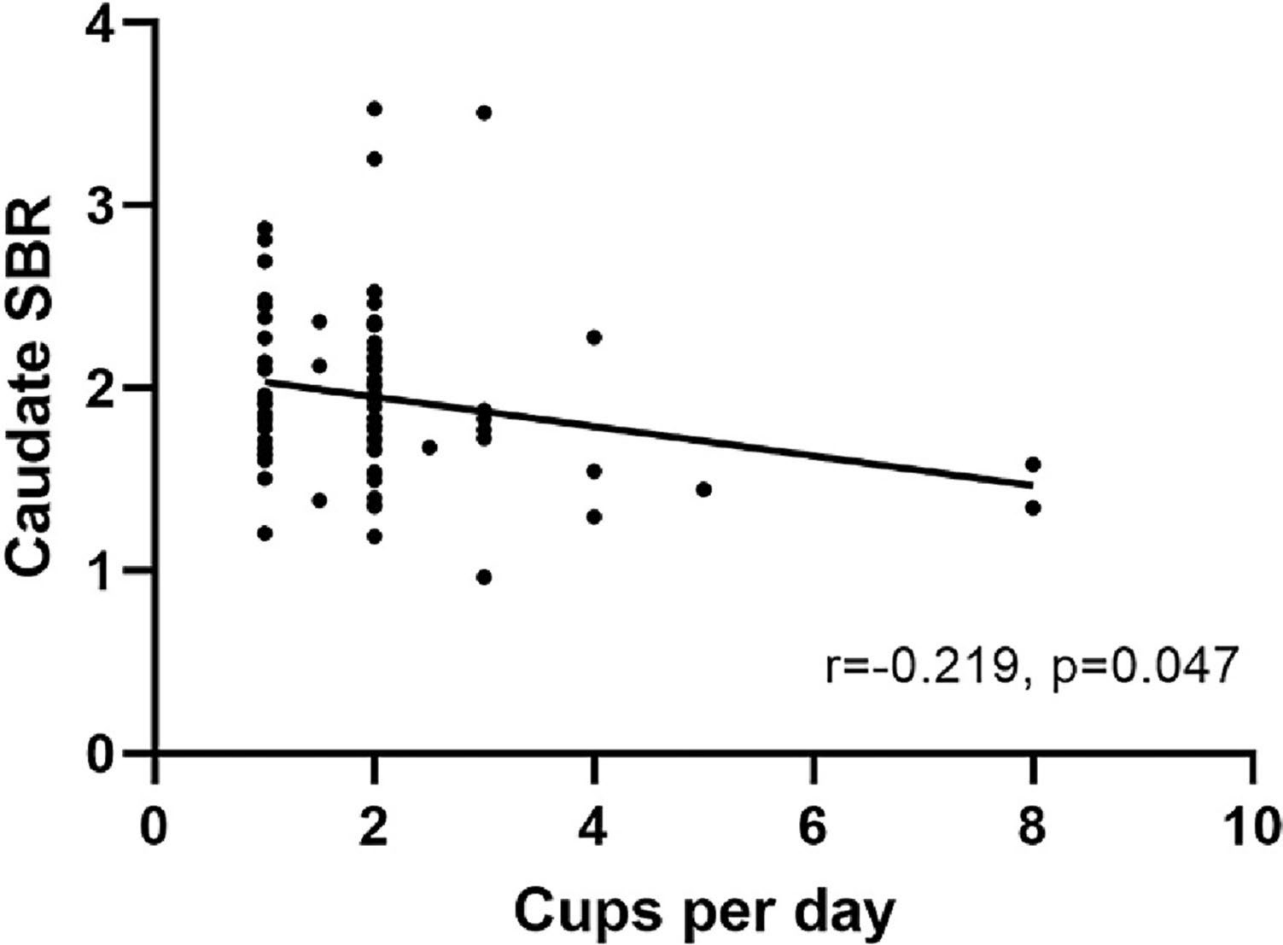
Correlation analysis showed that cups per day were negatively correlated with the caudate DAT availability in current consumers of PD patients. SBR, striatal binding ratios

PD patients had lower DAT availability in each striatal region compared to HC (*p* < 0.001) (Fig. 2, Additional file 1: Table S1). ANOVA analyses showed current coffee consumers had a tendency of lower DAT availability in the caudate than former/never coffee consumers in both PD patients and HC (Fig. 2, Table 2). In PD patients, there were significant differences in DAT availability in the caudate (*p* = 0.008, Bonferroni corrected) across three PD subgroups. Specifically, post hoc tests showed that current coffee consumers had significantly lower DAT availability in the caudate than former coffee consumers (*p* = 0.01) and never coffee consumers (*p* = 0.022). In HC, there were significant differences in DAT availability in the caudate (*p* = 0.031, Bonferroni uncorrected) across three HC subgroups. Specifically, post hoc tests showed that current coffee consumers had significantly lower DAT availability in the caudate than former coffee consumers (*p* = 0.022). The correlation analysis revealed that cups per day were negatively correlated with DAT availability in the caudate (*r* =  − 0.219, *p* = 0.047) in the PD-CC subgroup (Fig. 3, Additional file 1: Table S2).

**Table 2:** Dopamine transporter availability between subgroups of PD patients and healthy controls, respectively

	PD-CC (*n* = 88)	PD-FC (*n* = 18)	PD-NC (*n* = 32)	*p* values	HC-CC (*n* = 55)	HC-FC (*n* = 9)	HC-NC (*n* = 11)	*p* values
Caudate	1.95 ± 0.48	2.28 ± 0.63	2.19 ± 0.41	0.008**	2.99 ± 0.59	3.52 ± 0.69	3.34 ± 0.80	0.031*
Putamen	0.79 ± 0.27	0.84 ± 0.27	0.87 ± 0.22	0.317	2.17 ± 0.55	2.34 ± 0.51	2.59 ± 0.72	0.075

Bold values indicate significant differences

*PD-CC* current consumers of Parkinson’s disease, *PD-FC* former consumers of Parkinson’s Disease, *PD-NC* never consumers of Parkinson’s Disease, *HC-CC* current consumers of healthy controls, *HC-FC* former consumers of healthy controls, *HC-NC* never consumers of healthy controls

^*^Bonferroni uncorrected (*p* < 0.05)

^**^Bonferroni corrected (*p* < 0.05/4 = 0.0125)

### Independent contribution of coffee consumption to striatal DAT availability

The results of striatal DAT availability in multiple linear regression analyses of model 1 and model 2 are presented in Tables 3 and 4, respectively. Model 1 analysis showed negative associations between current coffee consumption and DAT availability in the caudate in PD patients (*p* = 0.004, Bonferroni corrected) and in HC (*p* = 0.011, Bonferroni corrected) (Table 3). In model 2, which was a stricter analysis that incorporated additional independent variables in PD patients including age at onset and disease duration, current coffee consumption remained independently and significantly negatively associated with DAT availability in the caudate (*p* = 0.003, Bonferroni corrected) (Table 4).

**Table 3:** Factors associated with dopamine transporter availability in each striatal subregion by a multiple linear regression analysis in PD patients and healthy controls

Striatal regions	Variables	Model 1 in PD (*n* = 138)			Model 1 in HC (*n* = 75)		
		*B* (SE)	95%CI	*p*	*B* (SE)	95%CI	*p*
Caudate	Age	− 0.009 (0.332)	(− 0.018, 0.000)	0.060	− 0.012 (0.007)	(− 0.026, 0.002)	0.097
	Gender	0.085 (0.005)	(− 0.096, 0.265)	0.355	0.304 (0.160)	(− 0.014, 0.622)	0.061
	Coffee consumption	− 0.258 (0.087)	(− 0.430, − 0.086)	**0.004**	− 0.423 (0.161)	(− 0.745, − 0.101)	**0.011**
	Smoking history	− 0.410 (0.289)	(− 0.982, 0.161)	0.158	− 0.600 (0.638)	(− 1.873, 0.672)	0.350
Putamen	Age	− 0.003 (0.002)	(− 0.008, 0.002)	0.256	− 0.015 (0.007)	(− 0.028, − 0.002)	0.029
	Gender	0.029 (0.049)	(− 0.068, 0.126)	0.553	0.154 (0.146)	(− 0.137, 0.445)	0.295
	Coffee consumption	− 0.064 (0.047)	(− 0.156, 0.029)	0.174	− 0.319 (0.148)	(− 0.614, − 0.024)	0.035
	Smoking history	− 0.149 (0.155)	(− 0.456, 0.158)	0.339	− 0.616 (0.584)	(− 1.782, 0.549)	0.295

Bold values indicate significant differences (Bonferroni corrected, *p* < 0.05/4 = 0.0125)

*PD* Parkinson’s disease, *HC* healthy controls, *B* unstandardized B, *SE* standard error, *CI* confidence interval

**Table 4:** Factors associated with dopamine transporter availability in each striatal subregion by a multiple linear regression analysis in PD patients

Striatal regions	Variables	Model 2 in PD (*n* = 138)		
		*B* (SE)	95%CI	*p*
Caudate	Age	− 0.039 (0.026)	(− 0.089, 0.012)	0.136
	Gender	0.085 (0.094)	(− 0.100, 0.270)	0.366
	Coffee consumption	− 0.262 (0.088)	(− 0.435, − 0.088)	**0.003**
	Smoking history	− 0.402 (0.292)	(− 0.979, 0.175)	0.170
	Age at onset	0.029 (0.025)	(− 0.020, 0.078)	0.238
	Disease duration	0.001 (0.006)	(− 0.011, 0.014)	0.829
Putamen	Age	− 0.014 (0.014)	(− 0.047, 0.022)	0.326
	Gender	0.034 (0.050)	(− 0.007, 0.243)	0.505
	Coffee consumption	− 0.067 (0.047)	(− 0.187, 0.047)	0.156
	Smoking history	− 0.139 (0.157)	(− 0.332, 0.446)	0.379
	Age at onset	0.011 (0.013)	(− 0.022, 0.044)	0.421
	Disease duration	− 0.001 (0.003)	(− 0.014, 0.003)	0.818

Bold values indicate significant differences (Bonferroni corrected, *p* < 0.05/2 = 0.025)

*PD* Parkinson’s disease, *B* unstandardized B, *SE* standard error, *CI* confidence interval

## Discussion

This study demonstrated that current coffee consumers had lower DAT availability in the caudate in PD patients and HC. Moreover, correlation analysis revealed that cups per day were negatively correlated with DAT availability in the caudate in PD-CC. In addition, after including the factors that may have influenced the loss of nigrostriatal dopaminergic neurons in multiple linear regression analyses, current coffee consumption remained an independent predictor of decreased DAT availability in the caudate in both PD patients and HC.

**Fig. 4:**
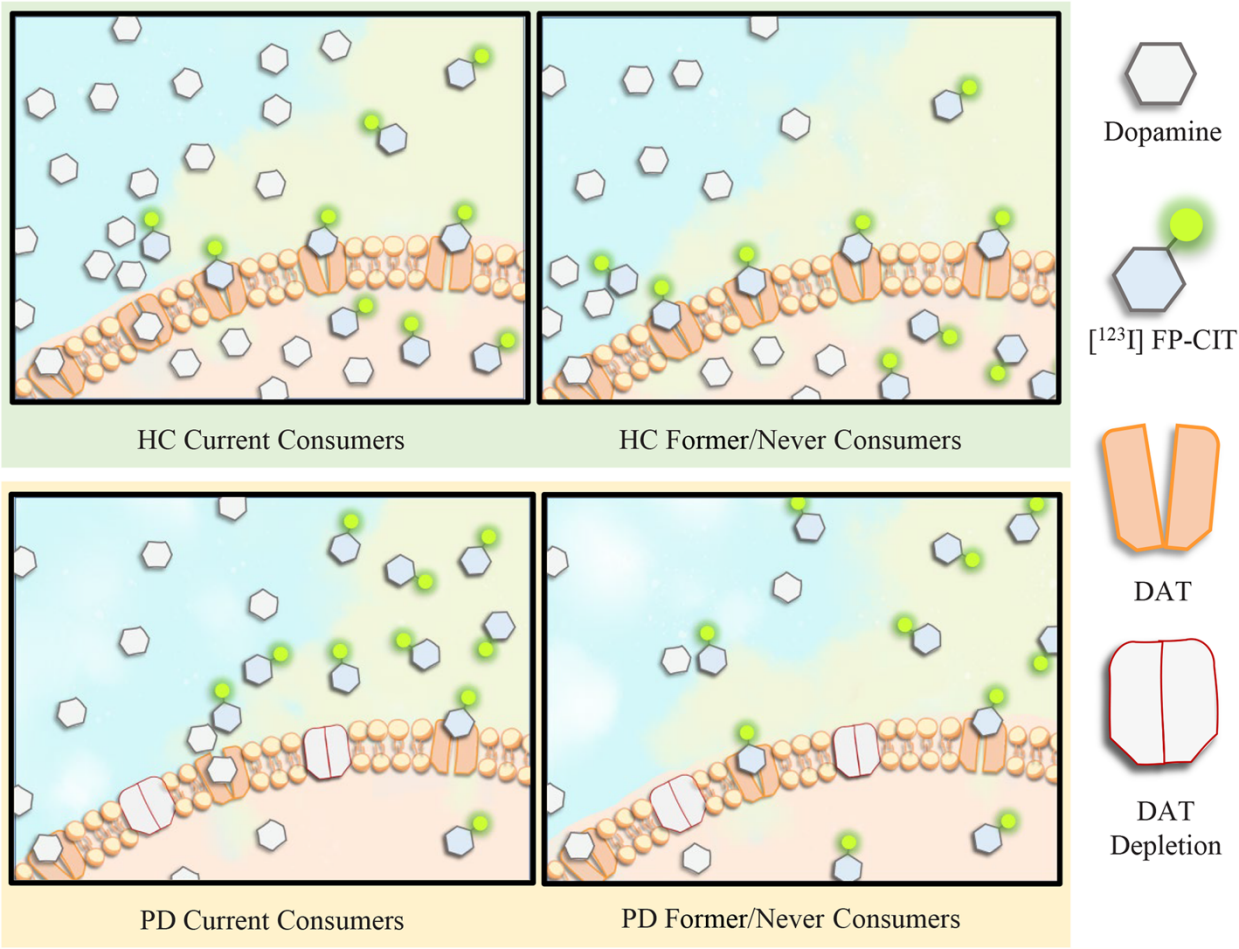
A proposed possible mechanism underlying the favorable implication of caffeine to PD pathologies. Caffeine enhances dopamine levels with a secondary reduced DAT availability in current coffee consumers compared to former/never coffee consumers in HC (**a**) and PD patients (**b**). The degeneration of dopaminergic neurons results in reduced DAT density and dopamine levels in PD patients (**b**) compared to HC (**a**). The inverse alteration of dopamine levels caused by PD pathologies and caffeine may be the reason for the favorable implication of caffeine to PD pathologies

Caffeine antagonizes adenosine receptors (i.e., A1, A2A, A3, and A2B), contributing to hyperexcitability of the central nervous system [1, 20]. A neuroprotective effect of caffeine is well documented in experimental PD models and is probably mediated by antagonizing adenosine receptors [21, 22]. Several experiments showed that caffeine pretreatment before 1-methyl-4-phenyl-1,2,3,6-tetrahydropyridine (MPTP) administration resisted dopamine depletion in a dose-dependent manner in mice [23, 24]. In MPTP mice, the residual dopamine level was 40% of control values with caffeine pretreatment (10 mg/kg), whereas it was 15% of control values without caffeine pretreatment [24]. At 20 mg/kg, caffeine pretreatment nearly reversed the dopamine depletion produced by MPTP in mice [24]. Furthermore, another experiment demonstrated that delayed caffeine administration could also reduce the loss of nigral dopamine cell bodies and block the nigral neurodegenerative process in rats [25]. DAT is expressed in the dopaminergic neurons and mediates the reuptake of free dopamine from the synaptic cleft back into the axonal button, thereby regulating dopamine levels [26]. This study demonstrated that current coffee consumers had lower DAT availability in the caudate in PD (*p* = 0.008, Bonferroni corrected) and HC (*p* = 0.031, Bonferroni uncorrected). In HC, the *p*-value (*p* = 0.031) did not pass the strict multiple comparison correction (Bonferroni correction). We speculate that this may be due to the small sample size of the HC group compared to the PD group (75 vs. 138 participants). Further multiple linear regression analyses supported this result that current coffee consumption was negatively associated with DAT availability in the caudate compared with former/never consumers in the HC group (similar to the results in the PD group). Caffeine releases the tonic inhibition of dopamine, boosts dopamine release, and induces a secondary reduced DAT availability [16, 27]. In PD, the loss of dopaminergic neurons caused by nigrostriatal degeneration results in a substantial reduction of the DAT density and dopamine levels [28]. Despite the similar reduced changes in striatal DAT availability caused by PD pathologies and caffeine, the inverse alteration of dopamine levels caused by PD pathologies and caffeine may provide the reason for the favorable implication of caffeine to PD pathologies (Fig. 4). Gigante et al. investigated the association between chronic coffee consumption and striatal DAT binding in PD patients, including 71 current coffee consumers and 12 never consumers. However, they reported negative results [29]. We believe that the discrepancies in the results may be related to the differences in sample size (83 vs. 138 PD participants), coffee consumption categorization (two categories vs. three categories), and analytical methods (caudate-based vs. alternative anatomical analyses). Furthermore, the present study is the first to detect a significant decrease in striatal DAT availability only in the current consumers but not the former consumers compared with never consumers, which suggests that effects of caffeine on striatal DAT availability may fade and disappear after quitting coffee consumption.

In addition, this study demonstrated that current coffee consumption was significantly negatively associated with DAT availability in the caudate but not in the putamen compared with former/never consumers in PD patients and HC. This finding indicates that caudate is more susceptible to the effects of coffee consumption compared with putamen. Using in vivo microdialysis, previous studies found caffeine increased striatal extracellular dopamine levels by antagonizing the adenosine A1 receptor [30, 31]. A molecular imaging study found that human caudate showed relative enrichment for adenosine A1 receptor compared to putamen (88 ± 4 vs. 83 ± 4) [32], which appears to be the main driver of caffeine facilitative effect on dopamine release [30]. In addition, caudate dopaminergic dysfunction is commonly seen in PD patients and plays a crucial role in the pathophysiology of parkinsonian symptoms such as cognitive impairment [33], depression [34], rapid eye movement sleep behavior disorder [35], and gait problems [36]. In addition, baseline caudate dopaminergic dysfunction is associated with an increased risk of developing cognitive impairment, depression, and gait problems over the follow-up [37]. At present, the clinical use of caffeine is not considered a contraindication to or even a confound of DAT imaging. Although current coffee consumption may cause a decrease in DAT availability, the descending range in DAT availability caused by caffeine is much lower than that caused by PD (Fig. 2). However, we believe that caffeine may lead to a false-positive diagnosis of some diseases for which the DAT availability decline is not yet obvious, such as prodromal PD. Therefore, whether coffee use should be banned before scanning is a question that needs further exploration, especially for some suspected diseases with decreased DAT availability in the caudate.

Although the present study showed chronic coffee consumption was associated with decreased striatal DAT availability, the retrospective study design in this study does not allow us to determine causality; we cannot determine whether the alterations of DAT availability predispose to coffee consumption initiation or whether chronic coffee consumption influences DAT availability. A case–control study investigated the association between cigarette smoking, alcohol drinking, coffee consumption, and PD risk [38]. The results showed coffee consumption was the highest factor in the reduction of PD risk. This strong inverse relationship remained stable when ever versus never coffee consumption was considered (OR = 0.16, *p* = 0.0001). This indicates that the proportion of never coffee consumers is higher in PD than in HC in that epidemiology study [38]. In the present study, the proportion of never coffee consumers in the PD group appears to be relatively higher than HC (23.2% vs. 14.7%). We compared the proportion of never coffee consumers between PD and HC using the chi-square test. However, there were no significant differences in the proportion of never coffee consumers between PD and HC (*p* = 0.139). It is still unclear whether predisposing factors, such as the genetic factors that make it easy to get into the habit of drinking coffee, the effects of chronic coffee consumption that lead to DAT availability abnormalities, or a combination of factors, are involved. Prospective longitudinal studies could answer this question. In the future, longitudinal imaging investigations should be designed to explore DAT availability changes before and after the initiation of coffee consumption.

In this study, the multiple linear regression analysis included smoking history (current vs. former/never smokers) as an independent variable. Unexpectedly, current smoking did not show any association with striatal DAT availability. Like coffee, cigarette smoking is a well-established protective factor for PD [39]. An inverse association between cigarette smoking and the risk of PD is well documented [40]. Several studies have found decreased striatal DAT availability in current smokers compared to non-smokers [41–43]. Nevertheless, after the onset, PD patients are able to quit smoking more easily than controls [44]. We believe that no significant results in our study may be mainly related to the small sample size of current smokers. In our study, only three PD patients were current smokers, and only one control was a current smoker.

The main strength of the present study is the large sample that allows testing different (current/former/never) coffee consumption effects on striatal DAT availability in both PD patients and HC. This study has several limitations. First, although several known confounding factors that significantly influence dopaminergic density were included in this analysis, it is possible that other lifestyle-related confounding factors may have altered the results. Second, similar to coffee, smoking is a well-established protective factor for PD [39]. However, PD patients are able to quit smoking more easily than healthy controls [44]. In this study, no significant association between current smoking and DAT may mainly be related to the small sample size of current smokers (only 3 PD patients). Third, although SPECT scans were begun 4 h (± 30 min) post-radiotracer injection according to the PPMI imaging protocol, information regarding the time of the last coffee cup consumed before SPECT scans was not available. Future studies should investigate the acute effects of coffee consumption on striatal DAT availability. Fourth, we investigated the association between caffeinated coffee consumption and striatal DAT availability in this study. Therefore, the findings of this study may not apply to decaffeinated coffee consumers. In addition to caffeine, there are many other potentially central nervous system active compounds in coffee that may alter DAT availability. Future studies should explore whether decaffeinated coffee consumption influences striatal DAT availability.

## Conclusions

In summary, this study demonstrated that current coffee consumption was associated with decreased striatal DAT availability in the caudate. Caffeine acts as a stimulant to boost dopamine release and induce a secondary reduced DAT availability, while the degeneration of dopaminergic neurons results in a substantial reduction of the DAT density and dopamine levels in PD. The inverse change of striatal dopamine levels caused by caffeine and PD pathologies may explain the favorable implication of caffeine to PD pathologies. Our study provides evidence for the favorable implication of caffeine to PD pathologies using human brain imaging in vivo. In addition, our study showed that reduced striatal DAT availability was detected in current coffee consumers but not in former coffee consumers compared with never coffee consumers, suggesting that caffeine’s effects on striatal DAT may fade and disappear after quitting coffee consumption.

## Supplementary Information

**Additional file 1:** **Table S1. **Dopamine transporter availability between PD patients and healthy controls. **Table S2. **Correlation analyses between coffee cups consumed per day and dopamine transporter availability in striatal subregions.

## Data Availability

The data that support the findings of this study are available from the PPMI website (http://www.ppmi-info.org/data). In addition, the data analyzed in this study are available from the corresponding author upon reasonable request.

## Abbreviations

ANOVA: One-way analysis of variance
DAT: Dopamine transporter
ESS: Epworth Sleepiness Scale
GDS: Geriatric Depression Scale
HC: Healthy controls
HC-CC: Current consumers of healthy controls
HC-FC: Former consumers of healthy controls
HC-NC: Never consumers of healthy controls
H-Y: Hoehn and Yahr
MDS-UPDRS: Movement Disorders Society Unified Parkinson’s Disease Rating Scale
MNI: Montreal Neurologic Institute
MOCA: Montreal Cognitive Assessment
MPTP: 1-Methyl-4-phenyl-1,2,3,6-tetrahydropyridine
PD: Parkinson’s disease
PD-CC: Current consumers of Parkinson’s disease patients
PD-FC: Former consumers of Parkinson’s disease patients
PD-NC: Never consumers of Parkinson’s disease patients
PPMI: Parkinson’s Progression Markers Initiative
QUIP: Questionnaire for Impulsive-Compulsive Disorders in Parkinson’s Disease
ROIs: Regions of interest
SBR: Striatal binding ratios
SCOPA-AUT: Scale for Outcomes for Parkinson’s Disease–autonomic function
SPECT: Single-photon emission computed tomography
STAI: State and Trait Anxiety Scale
UPSIT: University of Pennsylvania Smell Identification Test
