# Supplementary material for: Current coffee consumption is associated with decreased striatal dopamine transporter availability in Parkinson’s disease patients and healthy controls
Source: BMC Med. 2023 Jul 25;21:272. doi: 10.1186/s12916-023-02994-5 (PMC10369815; doi:10.1186/s12916-023-02994-5)
Supplement: Supplementary file 1 — Additional file 1: Table S1. Dopamine transporter availability between PD patients and healthy controls. Table S2. Correlation analyses between coffee cups consumed per day and dopamine transporter availability in striatal subregions. [file 12916_2023_2994_MOESM1_ESM.docx]

**Table S1. Dopamine transporter availability between PD patients and healthy controls.**

|  | PD | HC | p values |
| --- | --- | --- | --- |
|  | (n=138) | (n=75) |  |
| Caudate | 2.05±0.50 | 3.10±0.66 | **<0.001** |
| Putamen | 0.82±0.26 | 2.25±0.59 | **<0.001** |

Bold values indicate significant differences (Bonferroni corrected, p < 0.05/2=0.025). PD, Parkinson’s Disease; HC, healthy controls.

**Table S2. Correlation analyses between coffee cups consumed per day and dopamine transporter availability in striatal subregions.**

|  | PD-CC (n=85) | |  | PD-FC (n=18) | |  | HC-CC (n=53) | |  | HC-FC (n=9) | |
| --- | --- | --- | --- | --- | --- | --- | --- | --- | --- | --- | --- |
|  | Cups per day | |  | Cups per day | |  | Cups per day | |  | Cups per day | |
|  | r | p |  | r | p |  | r | p |  | r | p |
| Caudate | -0.219 | **0.047** |  | 0.017 | 0.951 |  | 0.100 | 0.483 |  | -0.291 | 0.527 |
| Putamen | -0.113 | 0.308 |  | 0.132 | 0.627 |  | 0.041 | 0.774 |  | -0.471 | 0.286 |

Bold values indicate significant differences (p < 0.05). PD-CC, current consumers of Parkinson’s Disease; PD-FC, former consumers of Parkinson’s Disease; HC-CC, current consumers of healthy controls; HC-FC, former consumers of healthy controls. In the correlation analyses, 85 PD-CC, 18 PD-FC, 53 HC-CC and 9 HC-FC who answered the ‘cfqa5day’ were included. ‘cfqa5day’ referred to the question “During the time you were regularly drinking caffeinated coffee, on average, about how many cups per day did you drink?”
